# Distribution, behavior, and erosion of uranium in vineyard soils

**DOI:** 10.1007/s11356-021-14381-9

**Published:** 2021-05-22

**Authors:** Daniel A. Campos, Sophia Blanché, Hermann F. Jungkunst, Allan Philippe

**Affiliations:** 1 iES Landau, Institute for Environmental Sciences, Group of Environmental and Soil Chemistry, University of Koblenz-Landau, Fortstraße 7, 76829 Landau in der Pfalz, Germany; 2 iES Landau, Institute for Environmental Sciences, Group of Geoecology & Physical Geography, University of Koblenz-Landau, Fortstraße 7, 76829 Landau in der Pfalz, Germany

**Keywords:** Mobility, Phosphate fertilizers, Viticulture, Colloidal transport, Soil structure, Prediction

## Abstract

Phosphate fertilization contributes to an input of uranium (U) in agricultural soils. Although its accumulation and fate in agricultural soils have been previously studied, its colloidal transport and accumulation along slopes through erosion have been studied to a lesser extent in viticulture soils. To bridge this gap, the contents and potential mobility of U were investigated in vineyard model soils in the Rhineland-Palatinate region, Germany. In addition to elevated U contents, U was expected to associate with colloids and subject to erosion, thus accumulating on slope foots and in soils with fine structure, and reflecting a greater variability. Moreover, another expectation was the favorable erosion/mobility of U in areas with greater carbonate content. This was tested in three regional locations, at different slope positions and through soil horizon depths, with a total of 57 soil samples. The results show that U concentrations (0.48–1.26 ppm) were slightly higher than proximal non-agricultural soils (0.50 ppm), quite homogenous along slope positions, and slightly higher in topsoils. Assuming a homogeneous fertilization, the vertical translocation of U in soil was most probably higher than along the slope by erosion. In addition, carbonate content and soil texture correlated with U concentrations, whereas other parameters such as organic carbon and iron contents did not. The central role of carbonate and soil texture for the prediction of U content was confirmed using decision trees and elastic net, although their limited prediction power suggests that a larger sample size with a larger range of U content is required to improve the accuracy. Overall, we did not observe neither U nor colloids accumulating on slope foots, thus suggesting that soils are aggregate-stable. Lastly, we suggested considering further soil parameters (e.g., Ca^2+^, phosphorus, alkali metals) in future works to improve our modelling approach. Overall, our results suggest U is fortunately immobile in the studied locations.

**Supplementary Information:**

The online version contains supplementary material available at 10.1007/s11356-021-14381-9.

## Introduction

Phosphate fertilizers result from the processing of phosphate rock that contains impurities such as uranium (U) mainly as uranyl (UO_2_^2+^) complex (Roessler 1990; Khater 2008). The continuous application of these fertilizers introduces large amounts of U, thus leading to its accumulation in soils (Takeda et al. 2006; Rogasik et al. 2008; Yamaguchi et al. 2009; Schipper et al. 2011; Wetterlind et al. 2012; Schnug and Haneklaus 2014; Bigalke et al. 2017; 2020). Recently, phosphate application resulted with the highest U input in agricultural land in comparison to other processes (e.g., manure application; Bigalke et al. 2020). Although there is currently limited information on U concentrations in viticulture soils, it has been reported that conventionally fertilized agricultural soils contain more U than organically cultivated vineyard soils (Steinmetz et al. 2017). In addition, U concentrations have been higher in topsoils in comparison to subsoils (e.g., Tarvainen et al. 2006; Utermann and Fuchs 2008; Steinmetz et al. 2017). Once mobilized, U from fertilized soils could leach into ground and surface water (Birke and Rauch 2008; Liesch and Hinrichsen 2015; Haneklaus et al. 2017), which is of concern considering experimental animal studies and human epidemiology (Brugge and Buchner 2011; Schnug and Lottermoser 2013).

The fate of U in soils depends mainly on its transformation processes, such as its binding/interactions to soil constituents through prevailing pH and Eh conditions (Langmuir 1978; Barnett et al. 2000; Echevarria et al. 2001; Igwe et al. 2005; Zhou and Gu 2005; Bednar et al. 2007; Gavrilescu et al. 2009; Schnug and Lottermoser 2013). In addition, Gueniot et al. (1988) pointed out that the geochemical behavior of U in soils is triggered by weathering processes (U accumulation and leaching) and the degree of soil evolution (U retention). Immobile U is mainly found in reducing environments as UO_2_, within the pH range of 4.0–7.5, mainly adsorbed to oxidized organic matter (OM), and precipitated on the surface of poorly or noncrystalline iron/aluminum (Fe/Al) minerals. Therefore, such minerals are considered important sinks for U (Takeda et al. 2006; Gavrilescu et al. 2009; Yamaguchi et al. 2009). Meanwhile, in oxidative environments, U mainly exists as UO_2_^2+^, and its sorption to negatively charged sites in soil components increases along with pH. The mobility of U, however, increases with the formation of soluble and negatively charged complexes with, for example, carbonate (Echevarria et al. 2001). Such stable and soluble complexes are considered more mobile and readily leached Sheppard and Evenden 1987; Rachkova et al. 2010; Vodyanitskii 2011; Cumberland et al. 2016). In a long-term study, Rogasik et al. (2008) observed that in German soils the estimated baseline values and accumulation rates of U were lower on very sandy soils than on soils with a higher clay and OM content. Similarly, Shahandeh and Hossner (2002) reported that the mobility and plant accumulation of U increase in soils with low adsorptive potential, in alkaline soils with carbonate minerals, and in the presence of chelates. Furthermore, Steinmetz et al. (2017) suggested that the affinity of U to Fe or water-extractable organic carbon may favor the mobility of U.

In addition, colloidal transport might play a considerable role on U mobilization. Colloids have a high sorption capacity due to their high specific surface areas and therefore can be effective sorbents of contaminants that are generally expected to be immobile (e.g., radionuclides, heavy metals, pesticides; de Jonge et al. 2004). Furthermore, mobile colloids act as a third phase, apart from the immobile solid constituents and the mobile aqueous phase, largely controlling the transport of pollutants in many environmental compartments (McCarthy and Zachara 1989; de Jonge et al. 2004; Baumann et al. 2006; Lead and Wilkinson 2006). Specifically, the mobility of colloidal U is favored by Fe, Al, minerals (e.g., clay), carbon-based particles (organic and inorganic) and macromolecules, pH-dependent sorption/desorption interactions, and the formation of alkali- and alkaline-earth uranates (Claveranne-Lamolère et al. 2009; Claveranne-Lamolère et al. 2011; Dreissig et al. 2011; Bots et al. 2014; Wang et al. 2014; Chen et al. 2018; Ge et al. 2018; Harguindeguy et al. 2018; Maria et al. 2020). For example, Yang et al. (2013) observed that the desorption of U from colloidal suspensions is influenced by interactions between metal/silicon oxides and humic acids, depending on their composition. Therefore, it is important to account for U distribution through soil particle size.

Another relevant factor for the fate of U in soils is erosion, which is influenced by several factors such as surface slope gradient, infiltration rate, rain intensity, and plant coverage (e.g., Ben-Hur and Agassi 1997; Fox and Bryan 2000; Liu et al. 2001). However, in areas with similar factors, soil mineralogy becomes a determining factor towards erosion. For example, kaolinitic soils have shown more aggregate stability than smectitic soils, and thus reflecting lower erosion (Singer 1994; Wakindiki and Ben-Hur 2002; Ben-Hur and Wakindiki 2004). The breakdown of particles depends on aggregate stability and soil properties (e.g., texture, organic matter content, clay/mineralogy) that influence soil dispersivity (Wakindiki and Ben-Hur 2002; Ben-Hur and Wakindiki 2004). Furthermore, the influence of slope gradient on infiltration rate varies, where infiltration rates decreased (Zaslavsky and Sinai 1981) or increased (Bradford and Huang 1992) along increasing slope gradients. In addition to soil mineralogy/texture, it is necessary to assess the variation of U concentrations in soils through several positions across the slope path.

Furthermore, the aforementioned studies have not proposed a unified model for U prediction through soil parameters. Therefore, the objectives of the present study were to determine whether the spatial distribution of U in vineyard soils is mainly due to erosion, which soil parameters are the most relevant predictors of U content, and whether U is related to smaller particle size fractions. Nonetheless, it is important to first specify what was defined as erosion in our study: the transport of specific soil components along the slope positions, reflected in a higher variability and a specific trend from the high slope position to the low slope position. This involved the measurement of U concentrations and other soil parameters in samples from three different vineyards at different positions relative to slope and across the soil profile at specific depths. Locations were selected based on contrasting soil texture, although their slope gradients are similar. Due to the proximity of the sampled locations, they are expected to have similar erosion processes (e.g., rainfall) and geological background. Lastly, it is assumed that there is a long-term homogeneous application of fertilizers over surfaces in each location, reflecting similar initial U contents along slope positions.

Based on the aforementioned literature, we hypothesized the following: first, we expected higher concentrations of U in topsoils than in deeper soils. Second, we expected a greater erosion in sites with higher carbonate and colloid contents. Third, we expected that due to erosion there are higher variations of U concentrations at the bottom of the slopes. Overall, the results and discussion were structured to first explore our data and identify soil characteristics per location. Thereafter, U variability was discussed to assess possible erosion patterns, followed by other similar patterns in other soil predictors. Furthermore, the regression analyses that identified the best predictors for U concentrations were discussed, and lastly these results in the light of previous studies propose useful predictors in future studies and, hence, improving the regression approach.

## Materials and methods

### Study area and sampling

Samples were obtained from three regional conventionally fertilized vineyard soils (Fig. S1) in the Rhineland-Palatinate region (Germany). We named each sampled area after its proximal village: “Böchingen”, “Edenkoben”, and “Ilbesheim” (Fig. S1). The location by Edenkoben was also part of a study from Fernández et al. (2015), whereas Ilbesheim was determined for this study during a field inspection. Sampling locations were selected randomly stratified showing differences in texture, although comparable in terms of slope (6–11%), their southward exposure, and their direct (relative) proximity to a road and a stream at the bottom of the slope.

For each sampling area, four horizon profiles were sampled from the surface down to 0.47 m at the upper (“Top”) and middle (“Middle”) slope positions of the hill. The soil turning point was identified in the profile at a depth of approximately 0.40 m. At the foot of the hill (“Base1”), samples were obtained only for the first 5 cm of the profile. Furthermore, a proximal sample to the foot of the hill, by the outer edge of the road, was obtained (“Base2”). The latter served to determine erosion residuals beyond the foot of the hill, assuming that highest U contents would accumulate there due to direct runoff. Four samples for thorough mixing, at each corresponding horizon depth and hill slope position (Top, Middle, Base1), were obtained on the northeast and southwest directions. Each sample replicate, at each horizon depth, weighted approximately between 145 and 210 g. A sampling scheme for depths in the Top and Middle slope positions is detailed on Fig. S2 (Supporting Information).

Nonetheless, soil samples from a proximal open area surrounded by forests, previously sampled by Fernández et al. (2015), were provided (Fig. S1). These samples were only analyzed for U content, serving as a reference for U content in proximal (non-agricultural) soils. The sampled areas comprise Quaternary formations, where the parent material is mainly Aeolian (Loess) sediment. The vineyard areas are classified with varying levels of erosion risk, ranging from “Low” to “Very High” throughout the sampling areas. Meanwhile, the erosion risk at the reference area is classified as “Very Low.” The geological information and erosion estimations were provided by the Office for Geology and Mining (*Landesamt für Geologie und Bergbau*) of Rheinland-Pfalz, Germany.

Furthermore, the application scheme of mixed NPK (nitrogen, phosphorus, and potassium) fertilizers was not reported. Unfortunately, general information on soil management and fertilizer application was limited. It was only informed that soils were not recently fertilized, up to a maximum of 5 years. In addition, the landowners did not communicate information on tillage, although it is known to be very seldom in the region and takes place about every 20 years. The overall sampling campaign took place from March until May of 2017, prior to the next fertilization period. To specify, the sampling campaign aimed to collect samples to determine the (final) distribution of U after any influence of erosion on U content, rather than the dynamic of U distribution after fertilizer application.

### Sample preparation

To assess the bulk density (BD) of single samples, the core cutter samples were weighted (bag weights were already determined a priori), and their individual water content was determined by drying 5 g of soil at 105°C. All sample replicates from each position, compass direction, and depth were thoroughly mixed and homogenized, resulting on 8 final samples for each Top and Middle position, 2 samples for the Base position, and 1 sample at the washout point in each location, with a total of 57 samples. Plant material was manually removed.

### Soil analysis

The concentration of U, Fe, Al, and Mn was determined following a reverse aqua regia microwave extraction method, assessing the fraction not included in (alumino) silicates. About 1 g of ground soil (previously sieved, 2 mm) was weighted in PTFE microwave vessels, followed by the addition of 6 mL HNO_3_ (suprapure 65%, Carl Roth, Karlsruhe, Germany) and 2 mL HCl (suprapure 35%, Carl Roth, Karlsruhe, Germany). Each sample was digested in duplicates. The vessels were lightly shook and left open for pre-digestion (10 min). Then, the vessels were closed tightly, and set in a microwave oven (Mars Xpress, CEM GmbH) for 40 min (incl. 10 min of heating time) at 160°C and 640 W (80% power). The extracts were transferred carefully into 50-mL centrifuge tubes using ultrapure water (resistivity = 18.2 MΩ·cm) to rinse and to fill the centrifuge tubes to a unified level of 45 mL. Samples were then centrifuged for 8 min at 3500 rpm (Universal 320, Hettich, Tuttlingen, Germany). Afterwards, 450 μL of supernatant was pipetted into 15-mL PP tubes and quantitatively diluted to 10 mL with ultrapure water for final analysis. Samples were measured for ^238^U via inductively coupled plasma mass spectrometry (Q-ICP-MS XSeries 2, Thermo Fisher Scientific, Dreieich, Germany), using Rhodium (^103^Rh, Fluka Analytical, Steinheim, Germany) as an internal standard. Al, Fe, and Mn were measured via inductively coupled plasma optical emission spectrometry (ICP-OES, Agilent 720 Series, Mulgrave, Australia) using the spectral lines at 396.15, 238.20, and 257.61 nm for quantification, respectively. For calibration, a U standard (Ultra Scientific, ICP-092; 1000 μg U/mL, 1% HNO_3_ preserved, Kingstown, RI, USA) was used for U quantification, and a multi-element standard (2% HNO_3_ preserved, Carl Roth, Karlsruhe, Germany) was used for Al, Fe, and Mn analysis. To address matrix effects, matrix-matched calibration curves were prepared, including acid blanks, by digesting the standards in the same acid mixture as for the samples. Lastly, instrument performance was monitored using the following reference materials: Tibet Sediment GBW 07327 (National Research Center for Certified Reference Materials, Beijing, China), Freshwater Sediment SRM 2703 (National Institute of Standards and Technology, Gaithersburg, MD, USA), and Lake Ontario water TMDA 52-4 (0.2% HNO_3_ preserved, Environment and Climate Change Canada). U recoveries were reported for TMDA 52.4 (109.4 ± 1.8%) and for Tibet Sediment (76 ± 1.4%) through eight replicates. It is important to precise that certified values in the sediment sample reflect its total content; however, as previously mentioned, the aqua regia digestion method does not dissolve silicates.

In order to provide a proxy for the content of colloids which can be suspended and potentially serve as a carrier for U, colloids were extracted following a method previously developed in our group (Philippe et al. 2018), and measured for turbidity which is assumed to be proportional to the total concentration of particles. For this, 6 mL of 0.1% Triton-X (Alfa Aesar, Karlsruhe, Germany) solution adjusted to pH 12 with NaOH (> 99%, Carl Roth, Karlsruhe, Germany) was added to 1 g of soil. The samples were vortexed for 10 s, and then sonicated for 15 min and vortexed again for 10 s. The suspension was centrifuged (Heraeus Multifuge 4KR, Thermo Scientific, Hanau, Germany) at 750 rpm (692 g) for 5 min, estimating a size threshold for particles < 1μm in diameter. Then, 1 mL of supernatant (in triplicates) was pipetted into a glass tube, diluted 1:6 with ultrapure water, and immediately measured for turbidity (HACH 2100AN Turbidimeter, Düsseldorf, Germany). Procedural blanks were also prepared following the same protocol.

The samples from the Middle slope position in each location were analyzed, as representative per location, with respect to their grain size analysis, pH, organic carbon (C_org_), and carbonate content. Gran size analysis was carried out following DIN ISO 11277:2002-08, determining the following fractions: coarse sand (2–0.6 mm), medium sand (0.6–0.2 mm), fine sand (0.2–0.06 mm), large silt (0.06–0.02 mm), medium silt (0.02–0.006 mm), fine silt (0.006–0.002 mm), and clay (< 0.002 mm). However, the results for the coarse sand fraction were not considered since their values were lower than 4% in all samples. In addition, we consolidated all silt fractions as one (“Silt”) since their values were highly correlated. Furthermore, C_org_ content was determined by dry combustion elemental analysis via CHNS Elementar Analyzer (Vario MICRO Cube, Elementar, Hanau, Germany), whereas carbonate content was determined after acid fumigation (Harris et al. 2001). Lastly, pH was determined following DIN 19684-1:1977-02 (0.01 M CaCl_2_).

### Data analysis

All statistical analyses were conducted using R Software (x64 3.6.3) and RStudio (Version 1.2.5042). Overall values for U and soil parameters (Fe, Al, Mn, turbidity, BD, gran size, C_org_, carbonate, and pH) for all locations are presented, and considered independent. U concentrations along slope positions in all locations (location-slope groups) were compared through an analysis of variances (ANOVA), and the significantly different group comparisons were identified through a Tukey honest significance test (HSD). These comparisons were conducted after testing for normality and variance homogeneity of U concentrations in location-slope groups. Correlations among measured soil parameters were determined using Spearman’s correlation (Table S1). The conducted principal component analysis (PCA) followed testing for multivariate normality and absence of orthogonal outliers using a quantile-quantile plot (Chi-square vs. Mahalanobis squared distances; Fig. S3) and orthogonal distances from all objects (Fig. S4), respectively. Hierarchical cluster analysis (package “factoextra”) followed a preliminary checking of correlation and STRESS between the original distance matrix (Euclidean distances) and average linkeages from the cluster matrix (Fig. S5) (Wildi 2010). Furthermore, Elastic Net (EN, package “glmnet”) and partial least square (PLS, package “pls”) were reported in terms of R^2^ along with regression coefficients (EN) and frequency of components (PLS) (Figs. S6 and S7). For regression models, 80% of the dataset was used for model training, while 20% was used as a test set for determining the accuracy. In order to evaluate the stability of the model, 50 random partitioning models were generated. Lastly, decision trees (DTs, package “dtree”) were conducted using the “bump” method (100 repetitions), and selecting the model with the lowest root-mean-square error.

## Results and discussion

### Soil characterization

**Fig. 1:**
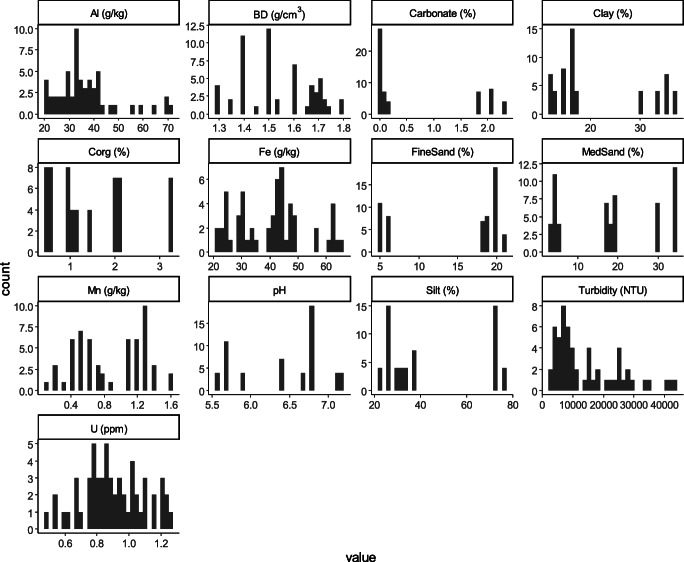
Density distributions for measured parameters in conventionally fertilized vineyard soils in the Rhineland-Palatinate region

The pH in soils ranges from slightly acidic to neutral (5.6 to 7.2). Furthermore, soils presented overall values for Al and Fe between 20.7–64.9 and 20.1–70.5 g/kg, respectively, and Mn contents in the range of 0.1–1.6 g/kg. Furthermore, extracted soil suspensions reflected turbidity values in the range of 3293–43841 NTU (Fig. 1).

**Fig. 2:**
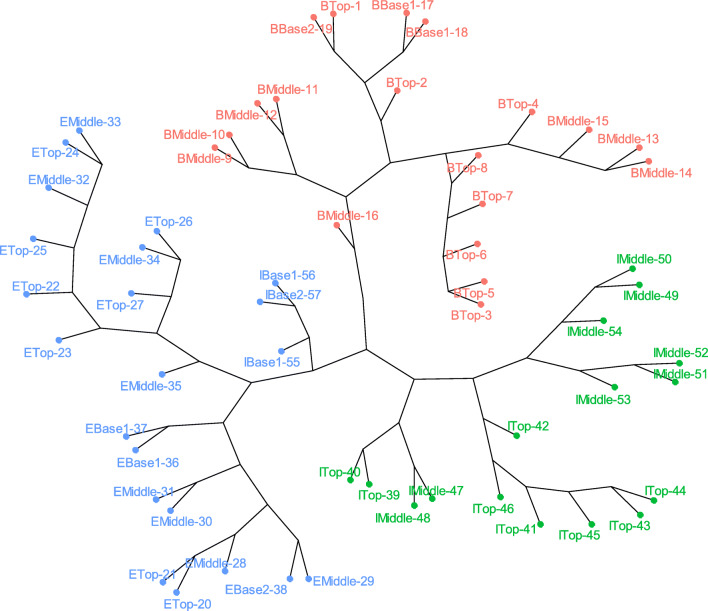
Hierarchical cluster analysis for sampled soils (n=57) in three vineyard locations: Böchingen (B, red), Edenkoben (E, green), and Ilbesheim (I, blue) at three slope positions (Top, Middle, Base1) and a proximal point from the bottom slope (Base2) in the Rhineland-Palatinate region. The colors correspond to the clusters obtained when setting the number of groups to 3

Nevertheless, each location has particular soil properties, as observed on the cluster analysis (Fig. 2) and the PCA biplot (Fig. 4). For example, soils in Böchingen had a silty-loam texture, as well as higher carbonate contents and greater silt fractions than in the other two locations. Meanwhile, soils in Ilbesheim had finer soil through a greater clay fraction and higher turbidity values. In addition, Ilbesheim soils had a clayey-loam texture. On the contrary, soils in Edenkoben had mainly a sandy-loam texture, although topsoil samples were classified as loamy.

As displayed in Fig. 2, there are sub-clusters within each location, which are mainly triggered by slope and depth positions in both Böchingen and Ilbesheim, although Base positions were clustered with a few sites from the Top (Böchingen) and Middle (Ilbesheim) slopes. In addition, similarities were observed despite profile depth. However, although grouped in a sub-cluster, Base slope samples showed more similarities with the overall Edenkoben cluster. However, sub-clusters in Edenkoben were not triggered by profile depth and slope position.

### Uranium concentrations

Measured U concentrations fell in an overall short range (0.48–1.26 ppm), and did not vary much per location and slope positions (Fig. S8). This value range falls within the estimated U concentrations in German topsoils (0.48–5.73 ppm) and subsoils (0.29–4.58 ppm) by Tarvainen et al. (2006). However, it has to be considered that only the Top and Middle slope positions were sampled along soil profile depths resulting in broader ranges compared to the Base positions. Furthermore, reference soil measurements (0.50 ppm) suggest that most samples in agricultural soils have U concentrations above natural backgrounds in proximal non-agricultural areas, despite the overall short range. HSD tests revealed that comparisons between groups within each location resulted in non-significant U concentration differences (p>0.05). In addition, it was observed that U concentrations were generally slightly higher in the uppermost profile layers, which was similarly observed in other studies (e.g., Takeda et al. 2006; Wetterlind et al. 2012; Steinmetz et al. 2017; Bigalke et al. 2020). However, it is possible that effects on leaching and accumulation of U (i.e., in respect to greater carbonate, clay, and/or C_org_ contents) are masked due to thorough mixing by deep tillage practices during preparation of new fields (Ziegler 2012). However, tillage in the studied region is seldom.

Nonetheless, such small differences along slope positions may reflect a homogenous application of fertilizers combined to a fast fixation of U compounds to soil components. Furthermore, U concentrations between Base1 and Base2 in all locations suggest that the erosion residual is very minimal. The fact that the position on the slope does not influence a variation of U concentrations suggests that erosion processes are not the main factor determining the accumulation of U, because this would be reflected in a relatively low concentration of U on top of the slope. Furthermore, the concentrations of other soil components supposed to be mobile (colloids, Fe, Mn, and aqua regia extractable Al) do not follow any clear pattern based on the position of the slope (Fig. S8), suggesting that erosion is not the main trigger for colloidal transport in our sampling sites. Therefore, soil properties are probably more relevant for predicting the presence of U than the position on the slope. In order to determine which parameter may be used to predict U concentrations, we carried out further multivariate analysis of our datasets.

### Soil predictors

**Fig. 3:**
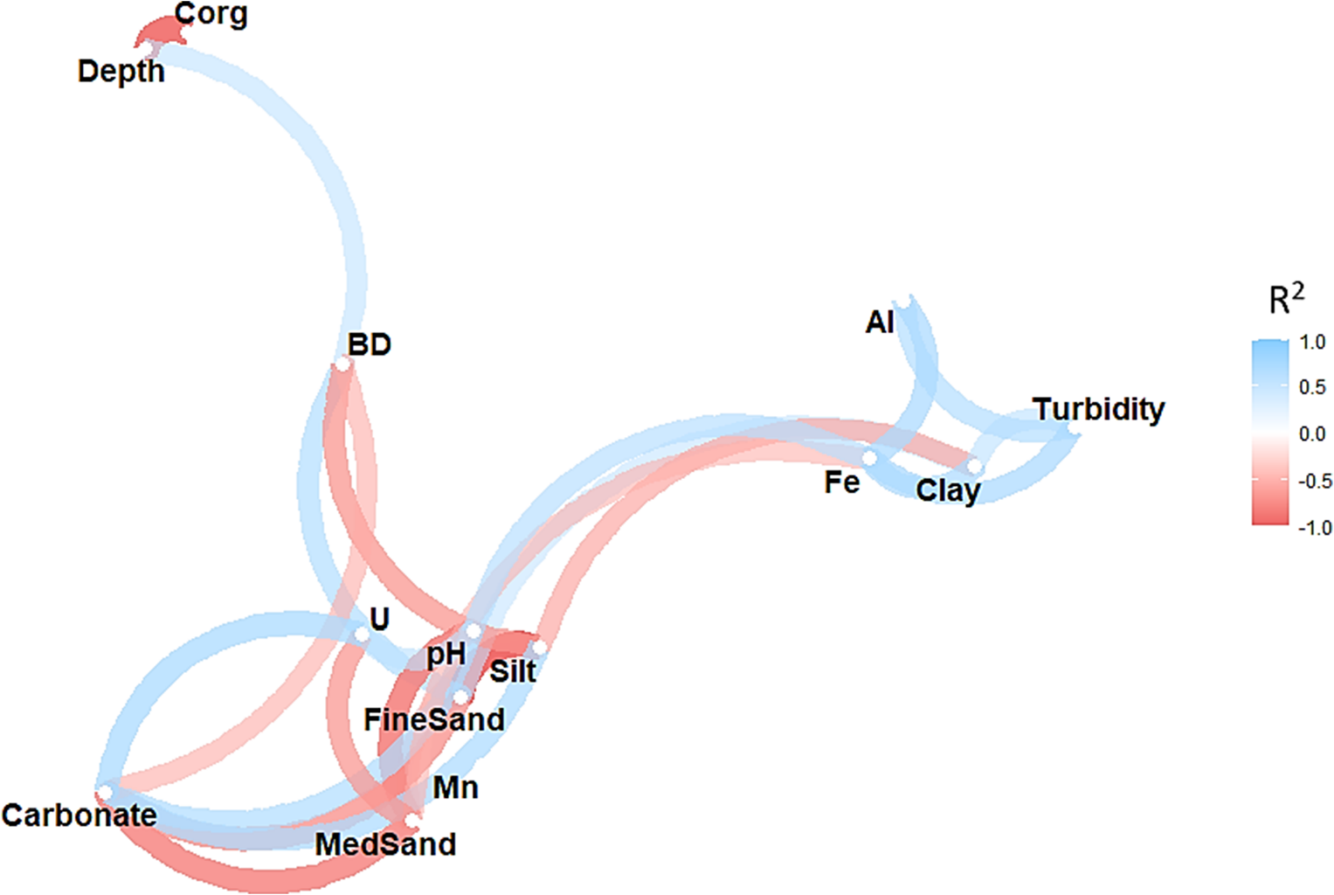
Network plot for measured soil parameters using Spearman correlations. Positive correlations between variables are connected with blue, whereas negative correlations with red. For clarity, only correlations with R^2^ > 0.5 are displayed. Color intensities and the spacing between variables correspond to the correlation magnitude. Detailed R^2^ values for each variable combination can be found in Table S1 (Supporting Information)

**Fig. 4:**
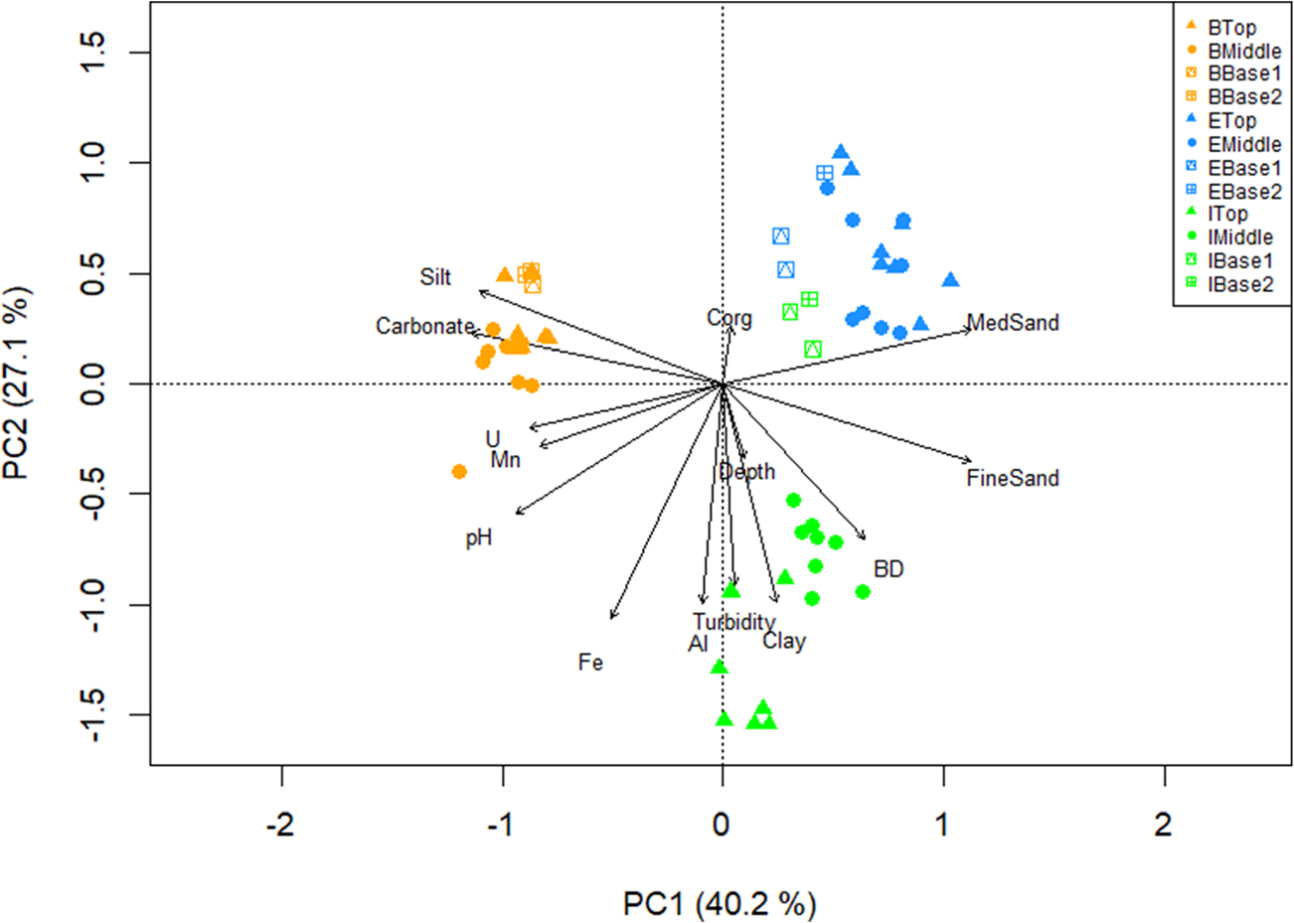
Principal component analysis (PCA) biplot on sampled sites (n = 57) from three agricultural locations: Böchingen (B), Edenkoben (E), and Ilbesheim (I) at three slope positions (Top, Middle, Base1) and a proximal point beyond Base1 (Base2). Arrows represent measured parameters on soil samples. The explained variances from the first two axes (PC1 and PC2) are displayed. BD: bulk density, C_org_: organic carbon, U: uranium, Fe: iron, Al: aluminum, Mn: manganese

The correlation map (Fig. 3) and the PCA biplot (Fig. 4) show that turbidity is linked to Fe and aqua regia extractable Al, thus suggesting that these elements are mainly present as colloids and serve as good proxies for erosion. However, unspecific variations of these parameters, as well as Mn, along slope positions were observed (Fig. S8), hence implying an overall minimal erosion. Unfortunately, these results cannot distinguish the respective effects of these variables. Other variables that are strongly linked are carbonate with soil texture (MedSand, FineSand, silt) and C_org_ with depth; however, it is not possible to assess their effects with the given sample set. Furthermore, based on our results, U seems to be linked to soil texture and carbonate.

Previous studies have shown relationships between U and the abundance of finer particles (Blanco-Rodríguez et al. 2008; Scheffer et al. 2010; Kumar et al. 2015), and with Al and Fe in agricultural soils (Yamaguchi et al. 2009). However, other studies have pointed out the importance of carbonates to determine U mobility (e.g., Echevarria et al. 2001; Bigalke et al. 2020). Therefore, in order to obtain a closer insight in the parameters influencing U concentrations, predictive models were built and analyzed.

### U prediction

**Fig. 5:**
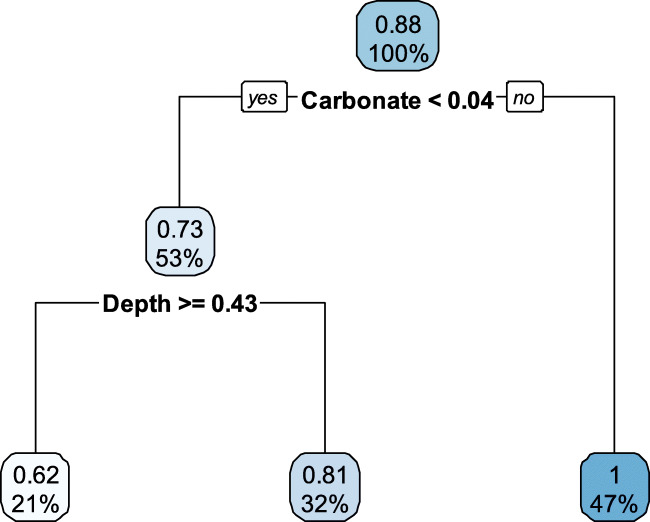
Selected decision tree (DT) for soil parameters on uranium contents. The selection was based on the resulted repetition with the lowest root-mean-square error (RMSE)

The conducted DTs resulted on carbonate and depth as the most decisive parameters as it could predict U content based on these two parameters only (Fig. 5). However, the moderate performance of the tree (R^2^ = 0.67) suggests that either our dataset is too small or the range of U content is not large enough. Similar conclusions can be drawn from the EN and PLS models, due to moderate R^2^ values with high deviations depending on the initial data-partition (Figs. S6 and S7). Despite the moderate performance of our model, it is important to mention that regression trees assess the effects of individual predictors through recursive partitioning, which is fit to subsets of both observations and predictors (Tomaschek et al. 2018). As previously mentioned, our model conducted 100 repetitions (“bump” method) and the tree with the lowest RMSE was selected. The results confirmed a higher response of U to carbonate than to other possibly linked variables (e.g., pH).

In addition, it was observed that carbonate and FineSand were most important in the EN model with highest regression coefficients in average, whereas others (i.e., depth and MedSand) showed lower coefficients and the remaining variables reflected minimal ones (Fig. S6). EN is an integral approach that combines both ridge regression and LASSO (regularized linear regression), which allows to differentiate the effects of collinear and variables and to shrink the effects of variables with low predictive power (Tomaschek et al. 2018). These results suggest that carbonate is a better predictor of U, followed by soil texture. Furthermore, such results may infer that, within our sampled locations, the soils in Böchingen can potentially favor the mobility of U.

Despite the moderate performance of our models, the predictability of U from carbonate has been suggested in other studies. For example, the sorption of U to minerals and soils is rather controlled by carbonate alkalinity than permanently charged clays (Duff and Amrhein 1996). Furthermore, the mobilization of U has been linked to the formation of soluble uranyl-carbonate complexes (Brookins 1988; Duff and Amrhein 1996; Gavrilescu et al. 2009), which increases with greater carbonate-to-phosphate ratios (Sanding and Bruno 1992). Furthermore, within the pH range of our studied soils, such carbonate complexes may be present (Gavrilescu et al. 2009). In addition, strong U carbonate complexation, through the formation of several ion pairs, enhances U mobilization (Duff and Amrhein 1996). Such complexation can increase mobilization by U desorption from binding sites of soil minerals and dissolution of mineral phases (Giammar and Hering 2001), although an increased dissolution of U-bearing particulates rather than mineral desorption has been observed (Elias et al. 2003).

Although other variables reflected very low coefficients, their relationship to U concentration and mobility has been previously reported. For example, U is more likely bound to extracted Fe-oxide than to OM and Mn-oxide fractions (Steinmetz et al. 2017). For instance, Roh et al. (2000) identified Fe-U oxide as one of the major U phases in studied soils. In addition, U-Fe-oxide and U-OM relationships reflect a greater immobilization of U (as uranyl phosphate) through diverse mechanisms (i.e., adsorption, heterogeneous nucleation, and co-precipitation; Giammar and Hering 2001); both U colloids and U-Fe/OM complexes may be as mobile as U carbonate associations (Wang et al. 2014). However, our results suggest that neither Fe nor C_org_ is a good predictor as carbonate (Fig. S6). Furthermore, a negative regression coefficient from depth could reflect the slightly greater U concentrations in topsoils, although the overall concentration range throughout soil depth is relatively minimal (Fig. S8).

PLS regression resulted in a slight improvement of the R^2^ median (0.59) compared to EN (0.55), which has been expected considering that the PLS approach, among its advantages over multiple regression (Cramer 1993), usually reduces the bias stronger than EN. By compromising principal components and regression, instead of the variable directly, the model flexibility increases and results in small bias (Filzmoser et al. 2009; Sarstedt et al. 2016). However, the improvement in terms of R^2^ is minimal and confirms that either the range of U content is too low or important parameters are missing. For instance, Kumar et al. (2015) considered the presence of minerals rich in alkali metals (i.e., Na^+^, K^+^) or Ca^2+^ influential on U sorption due to ion exchange. Furthermore, competition between U(IV) species and Ca^2+^-Mg^2+^ ions for surface sites has been observed (Duff and Amrhein 1996). Moreover, Zhou and Gu (2005) included Ca-U phosphate as one of the forms existing in investigated soils. Similarly, Roh et al. (2000) identified Ca-U carbonate, Ca-U silicate, and Ca-U phosphate as some of the major U phases in soils, being the latter the most common. Nonetheless, Steinmetz et al. (2017) observed a correlation between U and phosphorus, inferring the anthropogenic input of U through fertilizers. Overall, such parameters (phosphorus, Ca^2+^, Mg^2+^, alkali metals) should be considered in future modelling approaches to clarify their influence on U contents.

Interestingly, the low response of U to turbidity (Fig. S6) suggests that either U is not preferentially bound to colloids or these colloids are quite immobile. Associations of U to Fe oxides and organic matter, and thus its potential immobilization, has been previously reported (Hunter and Bertsch 1998; Yamaguchi et al. 2009; Steinmetz et al. 2017). Furthermore, U sorption on the surface of particles or attached as U phosphate colloids is possible. Greater sorption of U to particles has been observed to finer fractions and clays, as well as in greater presence of Fe (Kumar et al. 2015). In addition, sorption of U to silica colloids results in the formation of various complexes (Batuk et al. 2011). Furthermore, sorption of U to soil organic matter (e.g., humic acids) is dependent on pH, increasing when lower than 6 but forming soluble uranyl-humate complexes above 6 (Pompe et al. 1999). Nevertheless, it has been previously reported that humic acids hinder the sorption of U to soil particles because of binding site competition (Bednar et al. 2007). For our study, however, concrete conclusions on speciation are not possible with total content only. Our findings, though, rather suggest a general association of U to carbonates and silt fractions.

## Conclusion

Overall, spatial U variations along slope and location in our studied soils were minimal. Thus, it is inferred that the distribution of U in the locations and profile depth is governed by its vertical translocation and slight accumulation in topsoils, rather than erosion across the slope gradient. Although the distribution of U in soils is mainly homogeneous, its fixation is influenced by soil characteristics (i.e., carbonate and soil texture). However, our results did not show strong relationships between U and Fe, Al, and Mn, and extractable colloids as reported in previous studies. Furthermore, the prediction of U through soil parameters is possible, despite the low range of U concentrations and soil parameters covered in this study. This will help understand the mobilization of bioavailable U in vineyard soils. Furthermore, the introduction of missing parameters (e.g., Ca^2+^, phosphorus, alkali metals) in future studies can improve our modelling approach. Lastly, further investigation on the particle size distribution of U-bound particles should follow, including its speciation.

## Supplementary information

ESM 1 (PDF 1.97 mb)

## Data Availability

The dataset and R-script related to this study can be downloaded using the following link: 10.5281/zenodo.4290784.
