# Supplementary material for: Distribution, behavior, and erosion of uranium in vineyard soils
Source: Environ Sci Pollut Res Int. 2021 May 22;28(38):53181–92. doi: 10.1007/s11356-021-14381-9 (PMC8476358; doi:10.1007/s11356-021-14381-9)
Supplement: Supplementary file 1 — (PDF 1.97 mb) [file 11356_2021_14381_MOESM1_ESM.pdf]

Distribution, behavior, and erosion of uranium in vineyard soils

Daniel A. Campos[1]*, Sophia Blanché[1,2] Hermann F. Jungkunst[2], and Allan Philippe[1]*

* Corresponding authors (email: campos@uni-landau.de; philippe@uni-landau.de)

[1] iES Landau, Institute for Environmental Sciences, Group of Environmental and Soil Chemistry, University of Koblenz-Landau, Fortstraße 7, 76829, Landau in der Pfalz, Germany.
[2] iES Landau, Institute for Environmental Sciences, Group of Geoecology & Physical Geography, University of Koblenz-Landau, Fortstraße 7, 76829, Landau in der Pfalz, Germany.

Supporting Information

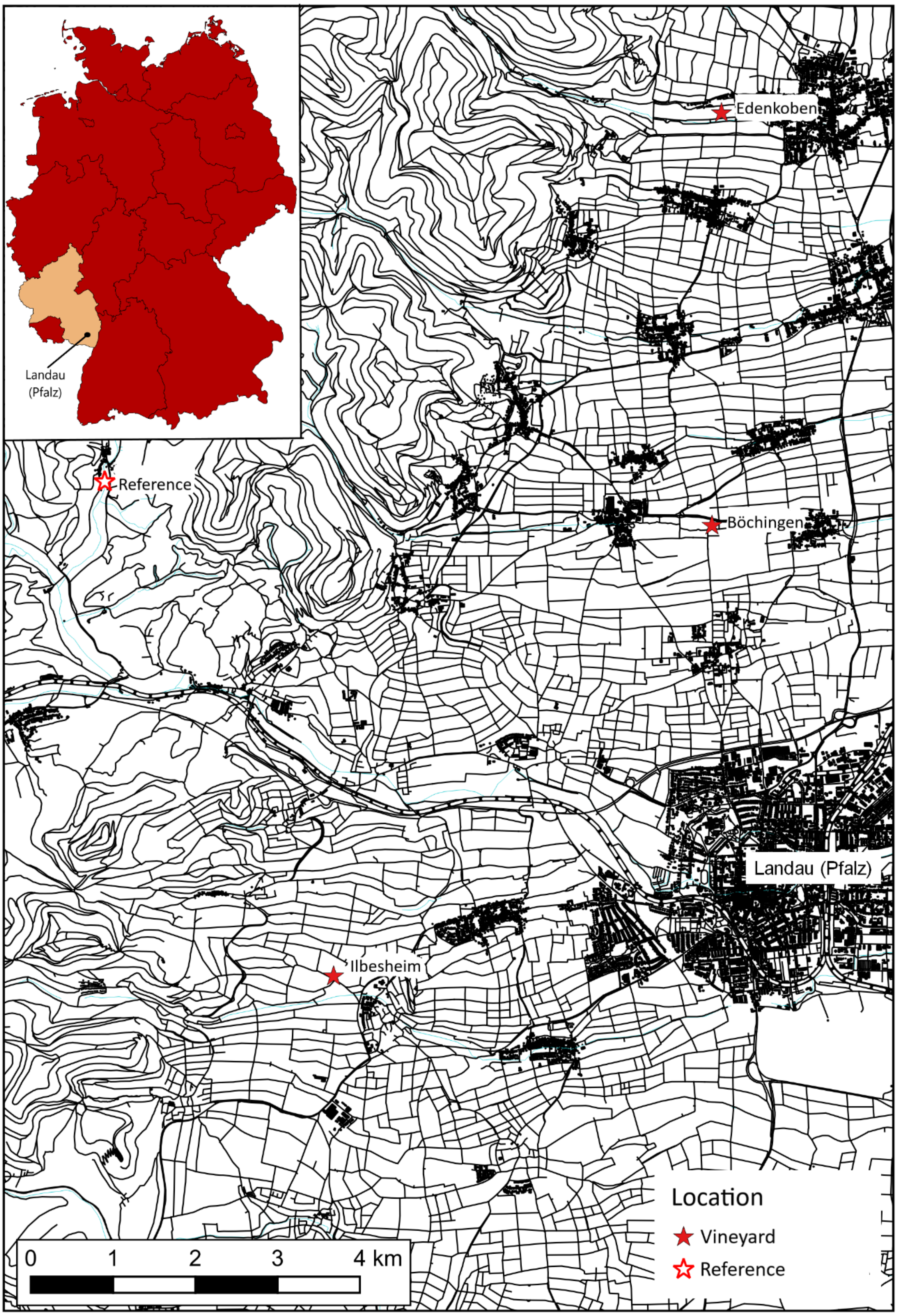

Figure S1. Sampled vineyards in the Rhineland-Palatinate region (cream), Germany. As a referential point, the city of Landau was marked and shown on the maps. Sampled areas include three vineyards named after its proximal village: “Böchingen”, “Edenkoben”, and “Ilbesheim”. A soil sample from a non-agricultural area (“Reference”, Fernandez et al. 2015) is also displayed. Regional streams/rivers are marked in blue. (Map databases/generators: Open street map, MapChart).

Table S1. Spearman's correlation matrix for soil parameters from vineyard soils in the Rhineland-Palatinate region. Correlations over ±0.7 are marked in bold. C_{org}: organic carbon, BD: bulk bensity, Al: aluminum, Fe: iron, Mn: manganese, U: uranium.

	**Depth**	**MedSand**	**FineSand**	**Silt**	**Clay**	**C_{org}**	**BD**	**pH**	**Carbonate**	**Turbidity**	**Al**	**Fe**	**Mn**	**U**
**Depth**	1.00	0.14	0.16	-0.17	0.06	**-0.93**	0.56	0.26	-0.19	-0.01	0.19	0.18	-0.01	-0.31
**MedSand**		1.00	0.55	-0.52	-0.30	-0.07	0.46	**-0.84**	**-0.81**	-0.21	-0.15	-0.53	-0.45	-0.69
**FineSand**			1.00	**-0.97**	0.54	-0.15	0.68	-0.37	**-0.73**	0.30	0.25	0.19	-0.39	-0.38
**Silt**				1.00	-0.61	0.16	**-0.71**	0.30	**0.71**	-0.40	-0.33	-0.28	0.34	0.39
**Clay**					1.00	-0.05	0.39	0.41	-0.06	0.58	0.43	**0.71**	0.01	0.21
**C_{org}**						1.00	-0.50	-0.34	0.21	-0.02	-0.26	-0.23	-0.05	0.25
**BD**							1.00	-0.13	-0.55	0.30	0.29	0.28	-0.26	-0.34
**pH**								1.00	0.69	0.28	0.27	0.64	0.43	0.59
**Carbonate**									1.00	0.01	-0.08	0.23	0.40	**0.73**
**Turbidity**										1.00	0.67	**0.72**	0.11	0.30
**Al**											1.00	**0.72**	0.42	0.22
**Fe**												1.00	0.41	0.47
**Mn**													1.00	0.42
**U**														1.00

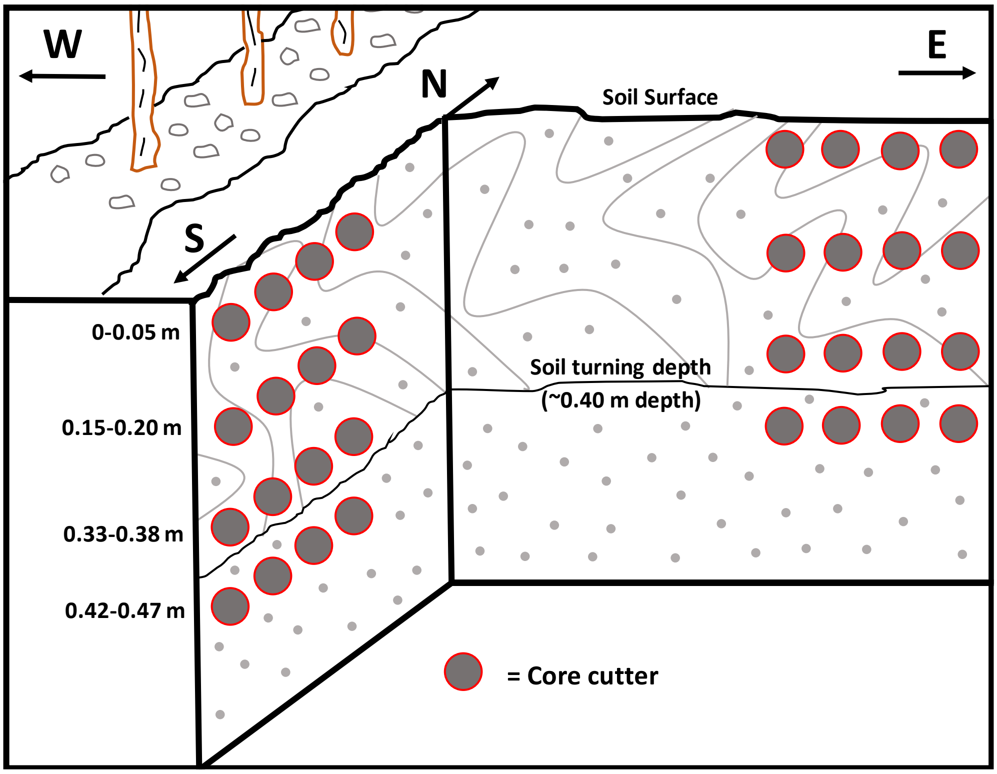

Figure S2: Scheme of collected soil sample replicates (prior to overall mixing) through profile depth and compass direction on Top and Middle slope positions. Meanwhile, samples from the Base (Base1 and Base2) were only obtained from the first 0.05 m depth level. Core cutter volume = 100 cm^3.

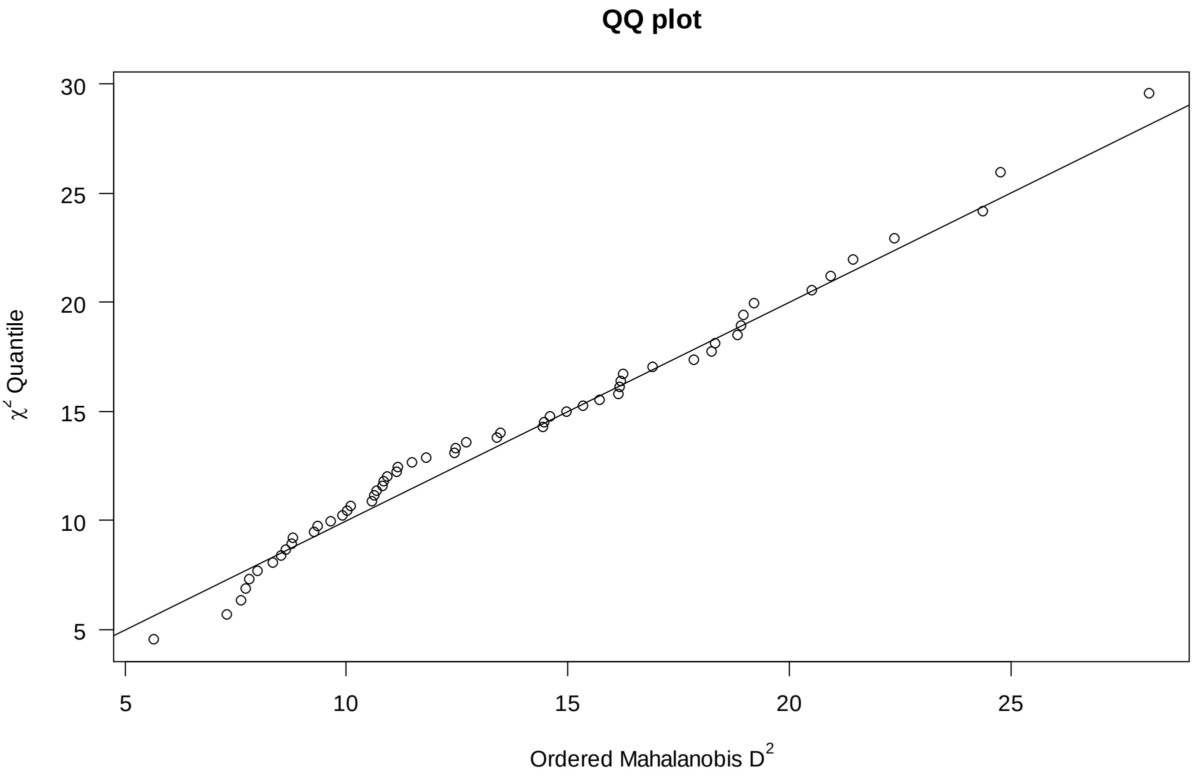

Figure S3. Multivariate normality and outlier assessment for PCA analysis using Chi-square (χ^2) quantile-quantile against Mahalanobis squared distances (D^2).

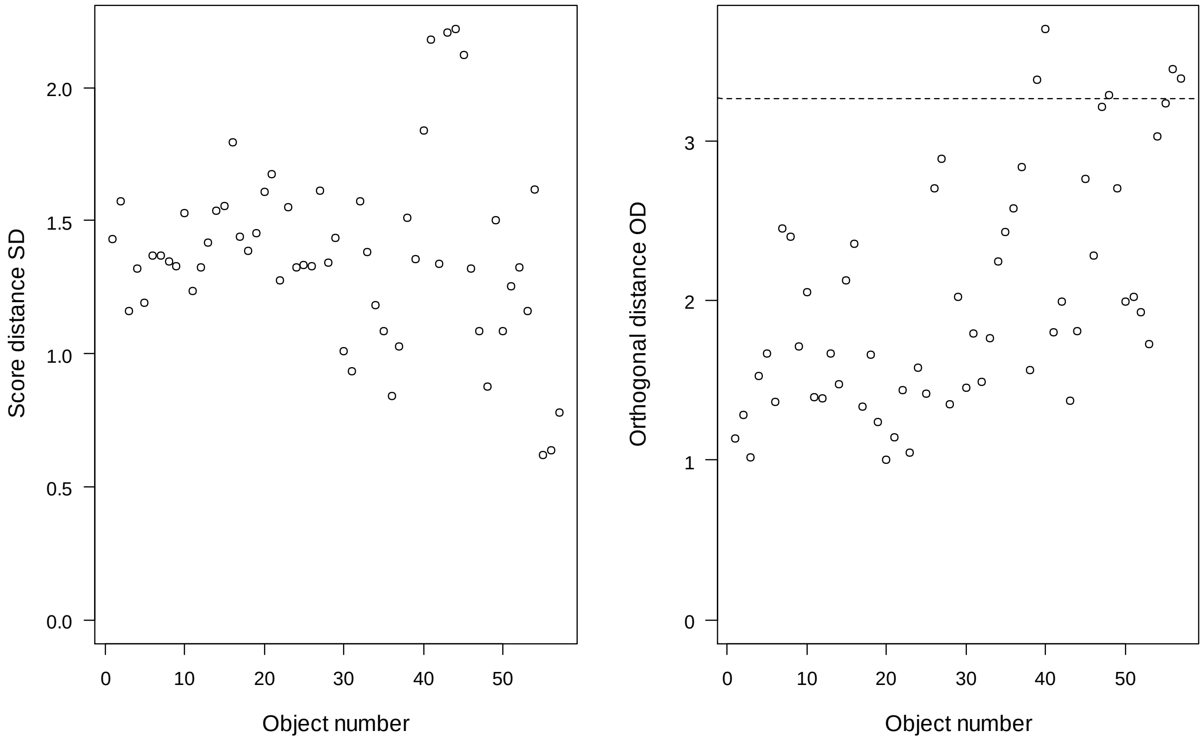

Figure S4. Outlier checking for PCA analyses using Score and Orthogonal distances through object number.

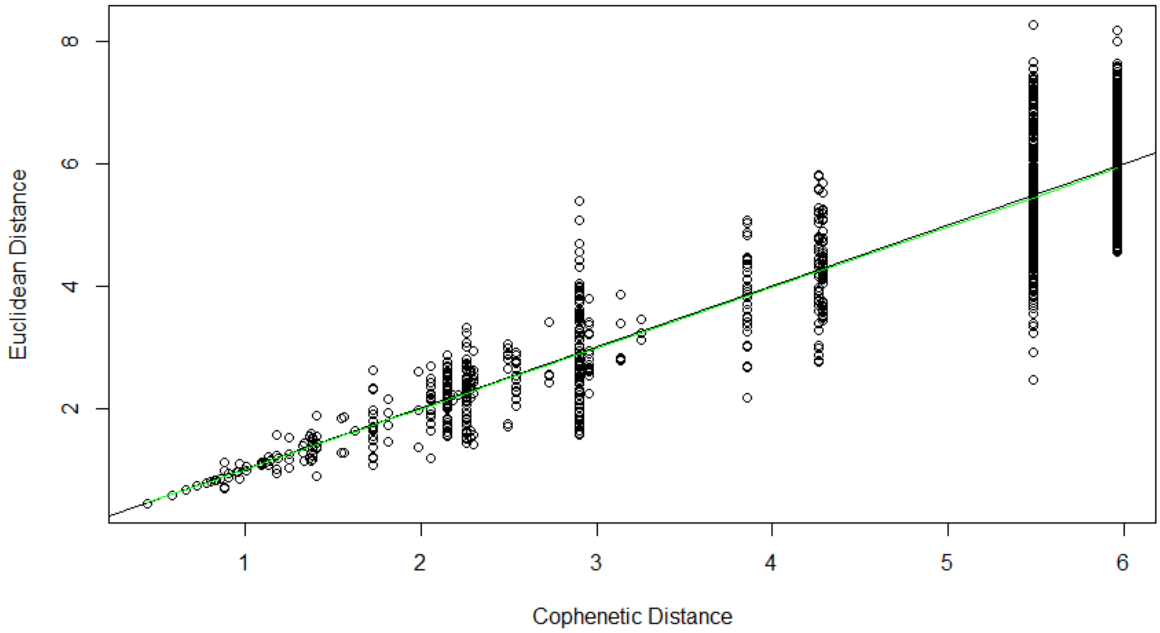

Figure S5. Comparison between original distances (Euclidean Distances) against Cophenetic Distances from the clustering analysis.

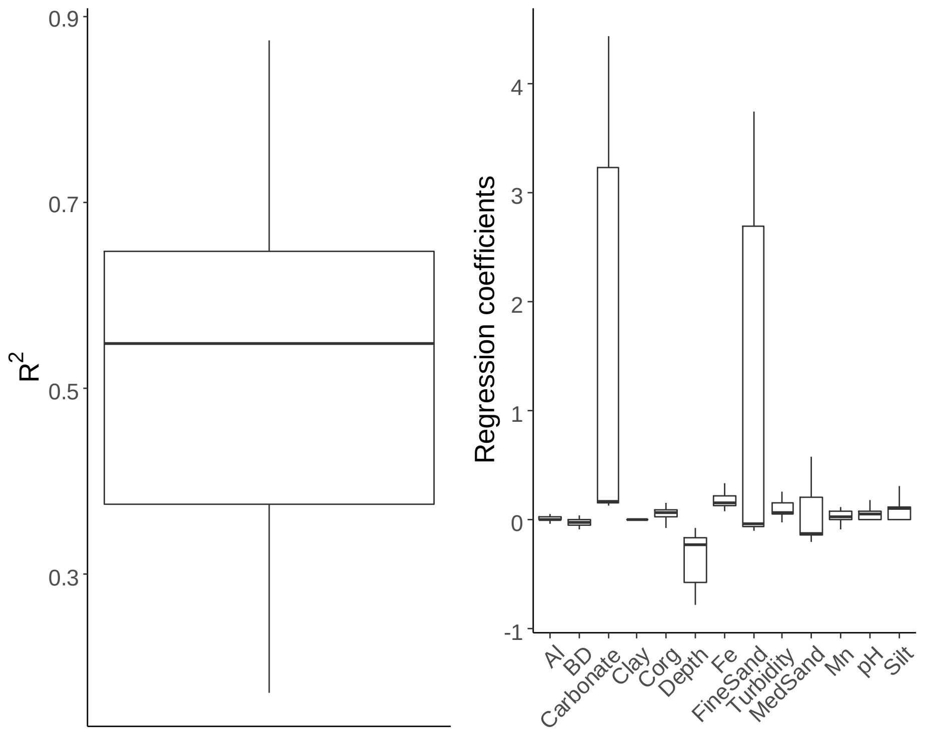

Figure S6. Evaluation of stability for the Elastic Net model though R^2 and regression coefficients. Error bars are included.

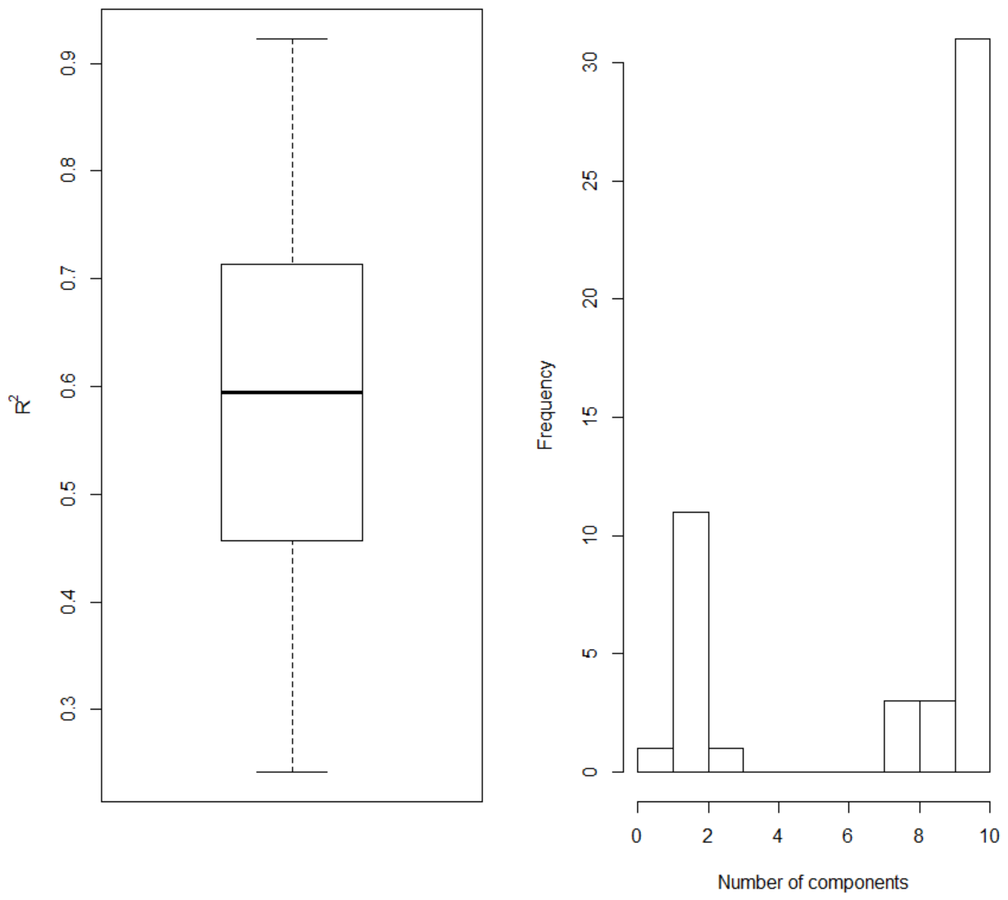

Figure S7. Evaluation of stability for the Partial Least Square model though R^2 and component frequencies.

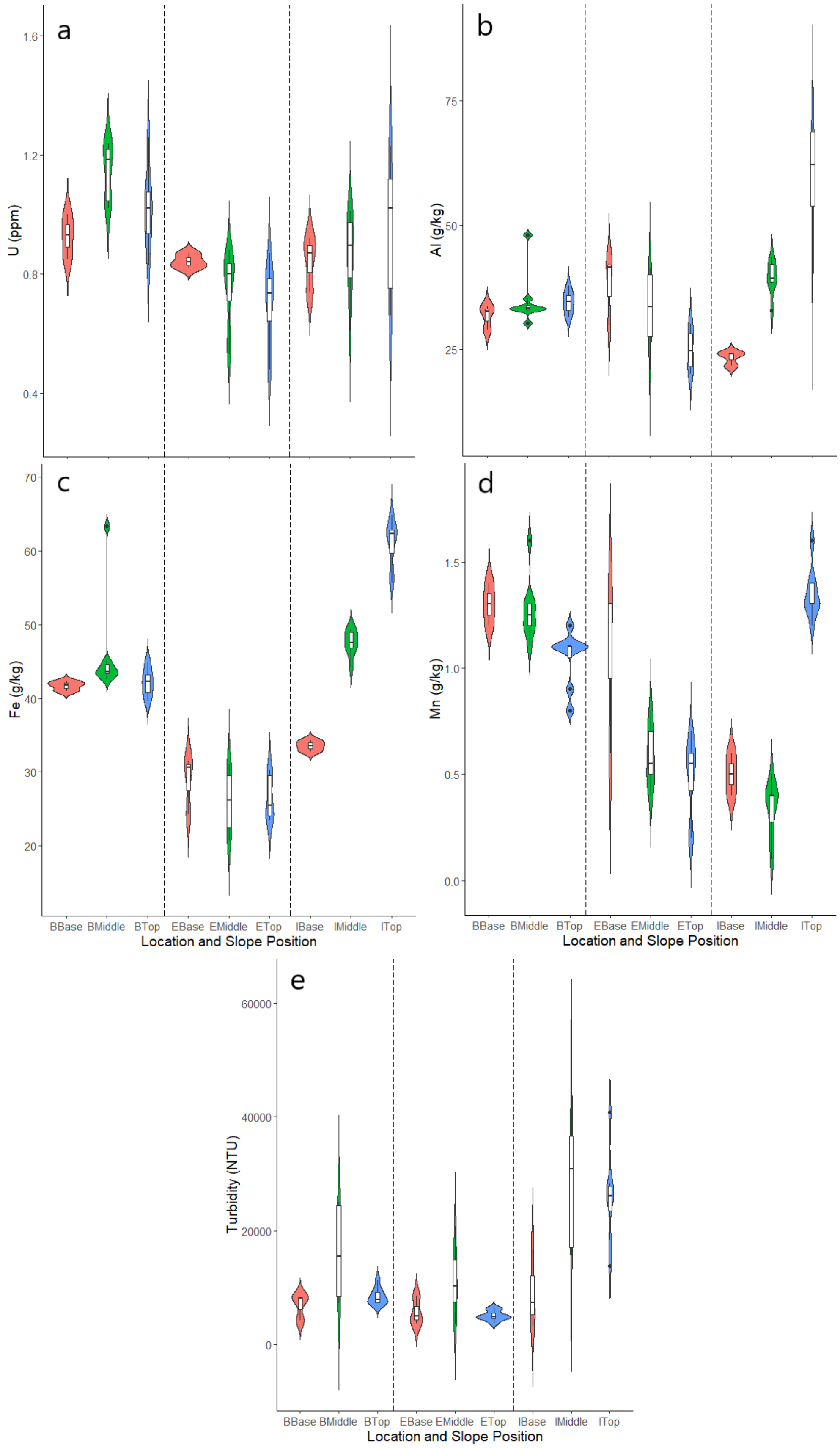

Figure S8. Violin-Box plot for **(a)** Uranium (U), **(b)** Aluminum (Al), **(c)** Iron (Fe), **(d)** Manganese (Mn), and **(e)** Turbidity measurements in soil samples (n = 57) from three viticulture locations: Böchingen (B, red), Edenkoben (E, green), and Ilbesheim (I, blue) at three slope positions: Top, Middle, and Base. Slope positions from Top and Middle include samples at different soil profile depths, whereas Base includes samples only from the topsoil. The Base slope position incorporates samples from both Base1 and Base2.

References

Fernández D, Voss K, Bundschuh M, Zubrod JP, Schäfer RB (2015) Effects of fungicides on decomposer communities and litter decomposition in vineyard streams. Sci Total Environ 533:40-48. https:doi.org/10.1016/j.scitotenv.2015.06.090
